# Comment on: Spectral domain optical coherence tomography predates fluorescein angiography in diagnosing central seruos chorioretinopathy

**DOI:** 10.4103/0301-4738.71688

**Published:** 2010

**Authors:** Kapil K Barange, Debarati Saha

**Affiliations:** Department of Ophthalmology, Gajra Raja Medical College, Gwalior, M.P.-474 009, India

Dear Editor,

This is with reference to the article by Gupta[1] claiming that “spectral domain HD OCT could pick up the presence of the sub retinal fluid and RPE changes even before they could manifest angiographically in CSCR”.

We submit as follows:

Both eyes at first presentation showed almost identical findings. Fundus fluorescein angiography (FFA) in both eyes revealed diffuse granular hyperfluorescence,[2] suggestive of chronic central serous chorioretinopathy (CSCR) [diffuse retinal pigmentary epitheliopathy (DRPE)].[2] Multiple pigment epithelial detachments seen on FFA in right eye (RE) (forme fruste)[3] and subretinal white deposits[3] in left eye (LE) further support the diagnosis. CSCR should have been diagnosed in both eyes (BE). CSCR can be asymptomatic.[4]

Considering the right eye at first presentation, line-scan optical coherence tomography (OCT) reveals one pigment epithelial detachment and neurosensory elevation. The retinal pigment epithelium (RPE) layer scan shows two pigment epithelial detachments and areas of irregularities. These findings can be seen in several other conditions such as presumed ocular histoplasmosis syndrome, idiopathic choroidal neovascularisation (CNV), Harada’s disease, posterior scleritis, etc. FFA at this point shows three distinct pigment epithelial detachments in addition to classical granular hyperfluorescence of chronic CSCR and gives a definite diagnosis. The authors have mentioned absence of smoke stack sign as an observation against the diagnosis of CSCR. Smoke stack sign is present in only 10% of acute cases.[5]

At second presentation, while the patient became symptomatic in RE, line-scan OCT did not show any increase in pigment epithelial detachment (PED) or neurosensory elevation. However, the single-layer RPE scan shows three points of pigment epithelial detachments, which were identified by FFA at the first presentation itself. Besides, FFA documents classical expanding dot sign, which corresponds to the patient becoming symptomatic due to acute attack. Obviously, FFA scores over OCT in early definitive diagnosis and follow-up of patients.

We wish to mention that OCT cannot predate any pathognomonic changes in CSCR. Sequential OCTs can only show the status of neurosensory and RPE detachments whereas FFA not only confirms the diagnosis but also helps in ruling out conditions that mimic CSCR, such as choroidal tumors and CNV that is not benign as CSCR.

The authors have advised comparison of Fig. 5c with Fig. 2d, where Fig. 5c belongs to the right eye and Fig. 2d belongs to the left eye.
